# Discovering dynamic brain networks from big data in rest and task

**DOI:** 10.1016/j.neuroimage.2017.06.077

**Published:** 2018-10-15

**Authors:** Diego Vidaurre, Romesh Abeysuriya, Robert Becker, Andrew J. Quinn, Fidel Alfaro-Almagro, Stephen M. Smith, Mark W. Woolrich

**Affiliations:** a Oxford Centre for Human Brain Activity (OHBA), University of Oxford, UK; b Oxford University Centre for Functional MRI of the Brain (FMRIB), University of Oxford, UK

## Abstract

Brain activity is a dynamic combination of the responses to sensory inputs and its own spontaneous processing. Consequently, such brain activity is continuously changing whether or not one is focusing on an externally imposed task. Previously, we have introduced an analysis method that allows us, using Hidden Markov Models (HMM), to model task or rest brain activity as a dynamic sequence of distinct brain networks, overcoming many of the limitations posed by sliding window approaches. Here, we present an advance that enables the HMM to handle very large amounts of data, making possible the inference of very reproducible and interpretable dynamic brain networks in a range of different datasets, including task, rest, MEG and fMRI, with potentially thousands of subjects. We anticipate that the generation of large and publicly available datasets from initiatives such as the Human Connectome Project and UK Biobank, in combination with computational methods that can work at this scale, will bring a breakthrough in our understanding of brain function in both health and disease.

## 1 Introduction

Understanding the nature of temporal dynamics of brain activity at a range of temporal and spatial scales is an important challenge in neuroscience. When studying task data, the aim is to discover the neural underpinnings and brain mechanisms elicited by the task, for which one relates the time course of the measured data to behaviour as comprehensively as possible. That is to say, we are interested in the dynamics evoked by the task. In this case, many repetitions of the same task are typically considered in the hope of characterising and interpreting the differences with respect to some baseline condition. Presumably, the brain adapts to the task at different time scales and in an online fashion, and we would like to capture these changes at as high a temporal resolution as the imaging modality will allow. When studying rest data, where the brain is not engaged in a predefined task, the brain will still process information dynamically, adapting its activity to the current perception of the environment combined with the products of its own spontaneous activity. In this case, then, we are interested in characterising the spontaneous dynamics. Either case, being able to characterise the temporal trajectories of whole-brain network activity at different time scales is, considering the complex and deeply integrated nature of the brain, crucial to understand the ultimate underpinnings of cognition (Buzsáki and Draguhn, 2004, Bressler and Menon, 2010).

**Fig. 1:**
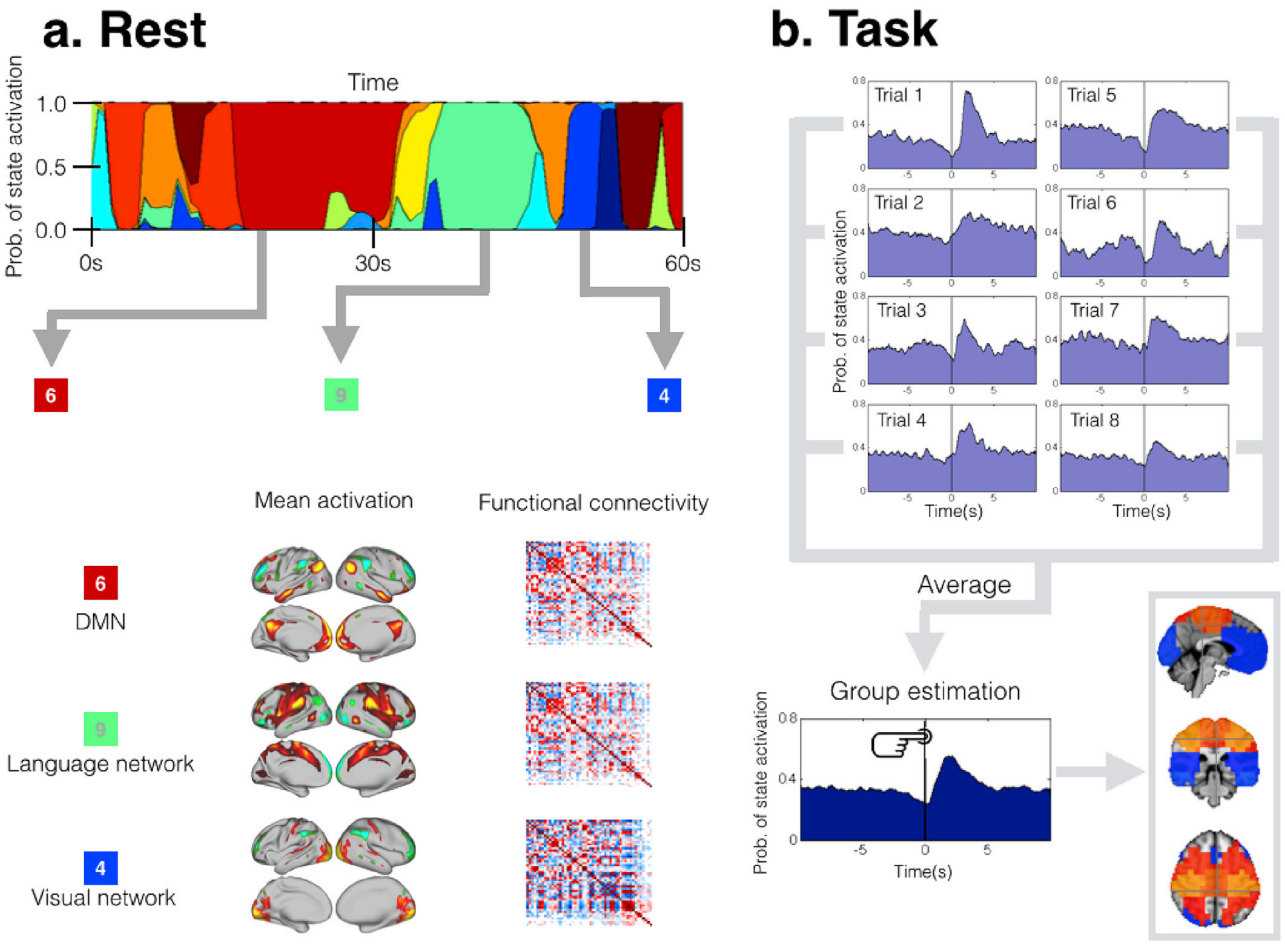
Scheme of HMM working on rest (**a**) and rest (**b**). In both cases, the HMM estimates several brain networks (or states) that are common to all subjects or trials, together with a specific state time courses for each subject which indicates when each state is active. In task, we can compute the state mean activation locked to the behavioural event, producing a *state evoked response*, which corresponds to a time-course of the proportion of trials for which subjects are in each state.

The most common analysis technique used for describing brain network dynamics in both task and rest is the use of sliding windows (Wendling et al., 2009, Allen et al., 2014). The sliding windows approach (and methods built upon it) suffers from a number of limitations (Hindriks et al., 2016) that can undermine any conclusions. In particular, they need a pre-specification of the time scale at which the neural processes of interest occur, i.e. the temporal width of the window. This choice is crucial and is a trade-off between two conflicting criteria: too long a window will miss fast dynamics, whereas too short a window will have insufficient data to provide a reliable network estimation. An alternative to the sliding window approach is the Hidden Markov Model (HMM), which can be used to describe brain activity as a dynamic sequence of discrete brain states, each characterised by a distinct pattern of network activity, including functional connectivity and/or spectral content (Baker et al., 2014, Vidaurre et al., 2016, Vidaurre et al., Woolrich). The HMM can be applied to task data to provide a rich description of the brain dynamics; for example, by estimating the HMM in a completely unsupervised way (i.e. with no knowledge of the task), and then post-hoc relating the HMM state sequence to the task timings, to reveal task-dependent functional connectivity dynamics (Vidaurre et al., 2016). This strategy can reveal task-related processes that are too fast to be seen by sliding window analyses (Vidaurre et al., 2016). The HMM can also be used on resting data to capture quasi-stationary states of activity that are consistently recurring over a population (Baker et al., 2014). This allows for the analysis of how certain dynamic properties vary across subjects, such as the transition probabilities between states or the differences of state occupancies (i.e. how much time is being spent in each state). An illustration of the HMM in both rest and task is presented in Fig. 1.

In the context of the HMM, increasing the amount of data can help to achieve richer and more robust conclusions about the dynamic nature of brain activity. In task, for example, having more trials will allow us to have a better understanding of the timing of brain activity in relation to the task and its trial-by-trial variability (which is due in part to noise but also to interesting cognitive processes such as learning). Fortunately, projects like the Human Connectome Project (HCP) (Van Essen et al., 2013) and UK Biobank (Sudlow et al., 2015) are recently producing an unprecedented amount of high quality data, with the number of subjects on the order of hundreds or thousands. However, group-level HMMs (run on data temporally concatenated over all subjects) are computationally expensive to train on such massive data sets. This problem is exacerbated if we use more complex HMM observation models (i.e. the model of the data produced for each HMM state), such as the use of a multivariate autoregressive model (MAR), designed to capture state-specific multi-region spectral information in electrophysiological data (Vidaurre et al., 2016).

In this paper, we propose an alternative to the standard HMM that uses a stochastic variational inference approach that can be applied to very large neuroimaging data sets, by greatly reducing its computational cost. The algorithm is generally applicable to the different instantiations of the HMM framework that are required for different data modalities. In the hope that it will be useful to other researchers, a Matlab toolbox implementing the algorithm has been publicly released.^1^ In the present work, we demonstrate the approach's performance on 820 subjects' resting-state fMRI data from the HCP, 5248 subjects' resting-state fMRI data from UK Biobank, and 52 subjects' MEG task-data from the HCP (this last dataset using a MAR observation model at high temporal resolution). Altogether, we use these examples along with simulated data to demonstrate that having a suitable computational method that scales well to large amounts of data can significantly enrich our description of dynamic brain activity.

## 2 Methods

### 2.1 The HMM and variational inference

The HMM is a family of models that can describe time series of data using a discrete number of states, all having the same probabilistic distributions but each having different distribution parameters. Thus, the states correspond to unique patterns of brain activity that recur in different parts of the time series. For each time point *t*, a state variable dictates the probability of each state being active at that moment. Fig. 1**a** exemplifies the model; on top, there are the state time courses, i.e. the values of the state variable across time; in the bottom, there is a graphical description of some states, in this case parametrised in terms of their mean activation level and functional connectivity. This general framework has different instantiations, depending on the choice of the observation model distribution. The most common variety of the HMM uses a multivariate Gaussian observation model, typically characterising the distribution of each state *k* by its mean ***μ***_*k*_ and covariance ***Σ***_*k*_ (Baker et al., 2014). It is important to bear in mind that our definition of a network is different from the activation maps that for example Independent Component Analysis (ICA) provides. The HMM Gaussian states contain not only an activation map (encoded by ***μ***_*k*_) but also a covariance matrix that can be interpreted as a network matrix of functional connectivity (as in e.g Fig. 1**a**). Another version of the HMM is the HMM-MAR, where the observation model is an autoregressive model and, thus, the states are defined and driven by their spectral signature (Vidaurre et al., 2016). In this case, both the amount of activity and connectivity are established as a function of frequency. A third possibility is the HMM-AR (HMM with autoregressive models), where the cross-channel interactions are not modelled. The AR is an intermediate point of model complexity between the Gaussian and the MAR models that keeps the channel-by-channel spectral information. Both the AR and the MAR have an important parameter: the model order, which controls the amount of detail in modelling the state spectra.

Whichever the chosen observation model distribution, an HMM generally comprises the description of the states, the state time courses (which determines the probability of each state to be active at each time point in the time series) and the transition probabilities between the states (i.e. the probability to transition from each state to each other state). Because here we run the HMM on all concatenated subjects' datasets, the states and the transition probabilities are defined at the group level; the state time courses are however particular to each subject - that is, states can come active at different moments for each subject. Since the probability distribution of each part of the model depends on all others, there is no closed-form solution available. A popular inference paradigm that assumes certain simplifications in the model is variational Bayes (Wainwright and Jordan, 2008), which has its roots in the field of statistical physics, and, earlier than that, in the calculus of variations developed in the 18th century by Euler and others mathematicians. The variational inference methodology introduces certain factorisations in the HMM probability distribution such that we can iterate through different group of parameters, leaving the remaining parameters fixed and thus reducing the computational burden. The goal is the minimisation of the so-called *free energy*, a quantity that includes the Kullback-Leibler divergence between the real and the factorised distributions and the entropy of the factorised distribution. The equations for the computation of the free energy in the context of the HMM can be found elsewhere (Vidaurre et al., 2016).

### 2.2 Stochastic variational inference for handling large data sets

The estimation of the observation model distribution for the Gaussian case implies the inversion of a *Q*-by-Q matrix per state, where *Q* is the number of channels or time series (e.g. brain areas); in the case of the MAR model, it requires the inversion of a *PQ*-by-*PQ* matrix, where *P* is the order of the MAR. In the standard variational inference approach, either case requires the entire data set to be loaded into memory. The estimation of the state time courses (for any HMM type of model, whether one uses variational inference or the maximum-likelihood approach) is based on the so-called Baum-Welch (also referred to as forward-backward) recursions (Baum et al., 1970), which, having computed the likelihood of each state at each time point, requires a complete sequential forward pass through the data followed by a complete backwards pass. Although this process is parallelizable across subjects, it can still be time consuming when the time series are long and/or the number of subjects is large. Therefore, standard variational inference for the HMM can be challenging for large data sets, because of (i) the memory required to estimate the observation models and (ii) the computation time taken by the estimation of the state time courses. In this paper, we make use of the principle of stochastic optimisation (Robbins and Monro, 1951) applied to variational inference (Hoffman et al., 2013) and apply it to the HMM, in order to develop a very efficient optimisation algorithm that addresses both computational difficulties, allowing for the estimation of the HMM on many subjects in just a few hours, and with modest memory requirements.

Standard variational inference guarantees the free energy to decrease at each iteration and, eventually, to converge. Stochastic variational inference instead performs a noisy and computationally cheap update at each iteration. Although those can occasionally lead to small free energy increments, they will typically improve the model. Over many iterations it is guaranteed to converge (Robbins and Monro, 1951, Hoffman et al., 2013). In this case, “noisy” means that we base each iteration on a random subset of *M* subjects: these are what we need to keep in memory to estimate what we refer to as the *interim* state observation models and for which we need to compute the state time courses. (Importantly, to obtain an interim state observation model, we must compute its parameters as though *N* subjects were actually used so that the estimation's properties mimic that of a standard variational step). This way, we have an interim estimation of the observation models, which (thanks to the additivity of the Gaussian and MAR distributions) can be linearly combined with the current estimation to form the new estimation. Such combination is parametrised by some scalar ρ such that

(1) $$\phi_{\mathrm{new}}=\left(1-\rho \right)\;\phi_{\mathrm{old}}+\rho \phi_{\mathrm{interim}}$$

where *ϕ*_*new*_, *ϕ*_*old*_ and *ϕ*_*interim*_ represent, respectively, the new, previous and interim posterior distributions of the observation models, and *ρ* decreases as the algorithm progresses, so that, at iteration *c* we have

(2) $$\rho =\rho \left(c\right)={\left(c+\alpha \right)}^{-\beta }$$

with *α* and β being some «delay» and «forget» parameters.

The estimation of the state transition probabilities is done exactly with little extra cost by keeping the sufficient statistics of the state time courses, Σ_t_ *Pr(s*_*t*_*) Pr(s*_*t-1*_*)*, where *s*_*t*_ represents the hidden state at time point *t*, and *Pr(s*_*t*_*)* represents the probability distribution of each state being active at *t*.

In Baum et al. (1970), the sampling of the subjects is purely random; here, we propose to (stochastically) promote those subjects that have been historically sampled fewer times. We do this through the following equation

(3) $$w_{i}=\tau^{{\text{r}}_{\text{i}}},$$

where *w*_*i*_ is the unnormalised probability of selecting subject *i*, *r*_*i*_ the number of times that subject *i* has been selected in previous iterations (scaled so that *min*_*i*_*(r*_*i*_*)* = *0*) and τ ≤ 1 is a parameter controlling how much we discourage subjects that have been frequently selected to be picked up at the current iteration.

The HMM optimisation is known to potentially suffer from having local minima. For this reason, and although stochastic inference can help to avoid flat local minima by virtue of the noisiness in the updates (Hoffman et al., 2013), the initialisation plays a crucial role because it can get the optimisation process away from poor regions of the parameter space. Hence, we need an initialisation mechanism that is computationally affordable in both time and memory use. The initialisation strategy that we propose here provides a reasonably good solution without being computationally expensive. In short, it consists of running separately the standard HMM inference on subsets of subjects and combining the results into a single solution using a matching algorithm. A detailed description is presented in the Supplementary material.

The algorithm is summarised as follows:

1. Initialise the observation models.
2. Repeat:
  a. Choose *M* subjects at random using probabilities, w_i_, computed as in (Eq. (3)).
  b. Compute the state time courses for the *M* subjects.
  c. Compute the interim state probability distribution using the *M* subjects as though we had *N* subjects.
  d. Perform an approximate update of the state probability distributions using (Eq. (1)).
  e. Perform an exact update of the state transition probabilities.
  f. Update *ρ* using (Eq. (2)).
  g. Compute the free energy.

Until the free energy converges.

In this paper, we have used *α* = *5*, β = *0.7*, and *τ* = *0.9*. There is a limited impact of the precise choice of *α,* β and τ. Of more importance is the choice of the value of *M*, which we varied depending on the data set (see below). As reported in Hoffman et al. (2013), very low values of M can have a negative effect, as the updates become too noisy; very large values can be computationally costly and typically do not bring much benefit. The chosen value is thus a trade-off.

Note that the general stochastic inference framework is the same for the Gaussian and the MAR state models, differing only in the particulars as to how to perform inference of the observation model parameters (step 2c). More comprehensive information can be found elsewhere about variational inference (Wainwright and Jordan, 2008), stochastic variational inference (Hoffman et al., 2013), the Hidden Markov model (Rabiner, 1989), and its applications to neuroimaging (Baker et al., 2014, Vidaurre et al., 2016, Vidaurre et al., Woolrich).

### 2.3 Simulated data

We first used synthetic signals to demonstrate the validity of the proposed stochastic inference approach. We generated two classes of signals using the HMM as a generative model. For the Gaussian observation model, we simulated 6 states, each with 10 regions and a randomly generated covariance matrix. For each subject, in order to verify that the stochastic algorithm produces similar results to the non-stochastic algorithm when there is some inter-subject variability, a small perturbation was applied to the covariance matrix by adding a second random matrix, sampled from the same distribution and scaled by a factor of 1 × 10^−2^. For the MAR observation model, on the other hand, we used 3 states, each with two channels/regions. These states were obtained from an actual HMM-MAR estimation on the task-MEG HCP data (described below), where we observed that two of the states (red and blue, see Results) were capturing the neural dynamics associated with the task, and the other state (green) corresponded to a baseline state. For both observation models, we simulated state time courses for 200 subjects, with 500 samples per subject.

### 2.4 fMRI data

We used resting-state fMRI data from N = 820 subjects from the HCP, all healthy adults (ages 22–35 years, 453 females) scanned on a 3 T Siemens connectome-Skyra. For each subject, four 15-min runs of fMRI time series data with temporal resolution 0.73 s and spatial resolution 2-mm isometric were available. The preprocessing pipeline is the same as in Smith et al. (2015): following artefact removal using FSL tools (Jenkinson et al., 2012), we used group spatial-ICA to obtain a “parcellation” of 50 components that covers both the cortical surfaces and the subcortical areas; then, we used this parcellation to project the fMRI data into 50-dimensional time series; such time series, of size (number of participants x number of scans x number of time points x number of ICA components = 820 × 4 x 1200 × 50), were standardised so that, for each scan, subject and ICA component, the data have mean 0 and standard deviation 1.

We also used resting-state fMRI data from the UK Biobank, a large-scale prospective epidemiological study aiming to allow the identification of risk factors and early biomarkers relating to many diseases (Sudlow et al., 2015, Miller et al., 2016, Alfaro-Almagro et al., 1000). We used the first public release containing 5847 resting-state subjects (aged 40–69 when recruited). The data was acquired in a 3 T Siemens Skyra using a 32-channel receive head coil with Multiband acceleration of 8x for 6 min and 10 s (490 timepoints), with temporal resolution of 0.735s, and spatial resolution of 2.4 mm isometric. Preprocessing was similar to that for HCP data, including motion correction, intensity normalisation, high-pass temporal filtering and EPI unwarping (Alfaro-Almagro et al., 1000). We then use ICA (Beckmann and Smith, 2004) followed by automated classification and removal of the artefact components (Griffanti et al., 2014). Group-ICA was then run using the first 5248 subjects' cleaned data, resulting in 100 group-level components, of which 55 were manually classified as non-artefact and used for the HMM analyses. Dual regression analysis (Beckmann et al., 2009) is performed to obtain subject-specific node timeseries associated with the group-level parcels, for feeding into the HMM analyses.

### 2.5 MEG data

We used 52 MEG subjects from the HCP having both resting state and the motor task data available (Larson-Priora et al., 2013). Resting state data consisted three sessions (6 min each, eyes open), and the examined task data has two sessions of 14min each. The chosen task sessions consisted of blocks of moving either left or right hand and feet respectively. Here, for simplicity, we used the right hand moves only. We used the preprocessing pipelines offered by the HCP consortium, removing bad channels, segments and bad independent components from task and rest data.^2^ After bandpass filtering (1–48 Hz), the MEG data were LCMV-beamformed (Woolrich et al., 2011). Using the AAL atlas, we considered the two parcels representing left and right precentral gyrus, and use PCA to extract the first principal component from each one. The motor task was a simple localizer task that included right hand movements; data epochs were time-locked to EMG onset and downsampled to 100 Hz. For the resting-state data, artificial epochs of the same size as the task epochs were uniformly spread throughout the session. In both the rest and task data, the resulting parcels were then sign-flipped, if necessary, to match the corresponding MEG sensor motor-related evoked fields with same polarity across all subjects (Vidaurre et al., 2016). Parcel time-series were also normalized before being subject to HMM analysis, such that they have mean equal to zero and standard deviation equal to one for all subjects separately.

### 2.6 Available software

The HMM is available in both the standard and the stochastic inference versions as a Matlab toolbox in a public repository.^1^ Currently, Gaussian, MAR and AR observation models are available, with the option of working in PCA space to control the dimensionality. This is particularly useful in the case of the MAR model, where the number of model parameters rapidly (quadratically) increases with the number of channels. The toolbox also offers some data preprocessing utilities and two different ways to compute the state spectra once the HMM has been estimated: one makes use of a weighted version of the multitaper introduced in Vidaurre et al. (2016), and the other uses the MAR or AR parameters directly.

## 3 Results

### 3.1 Simulated data

**Fig. 2:**
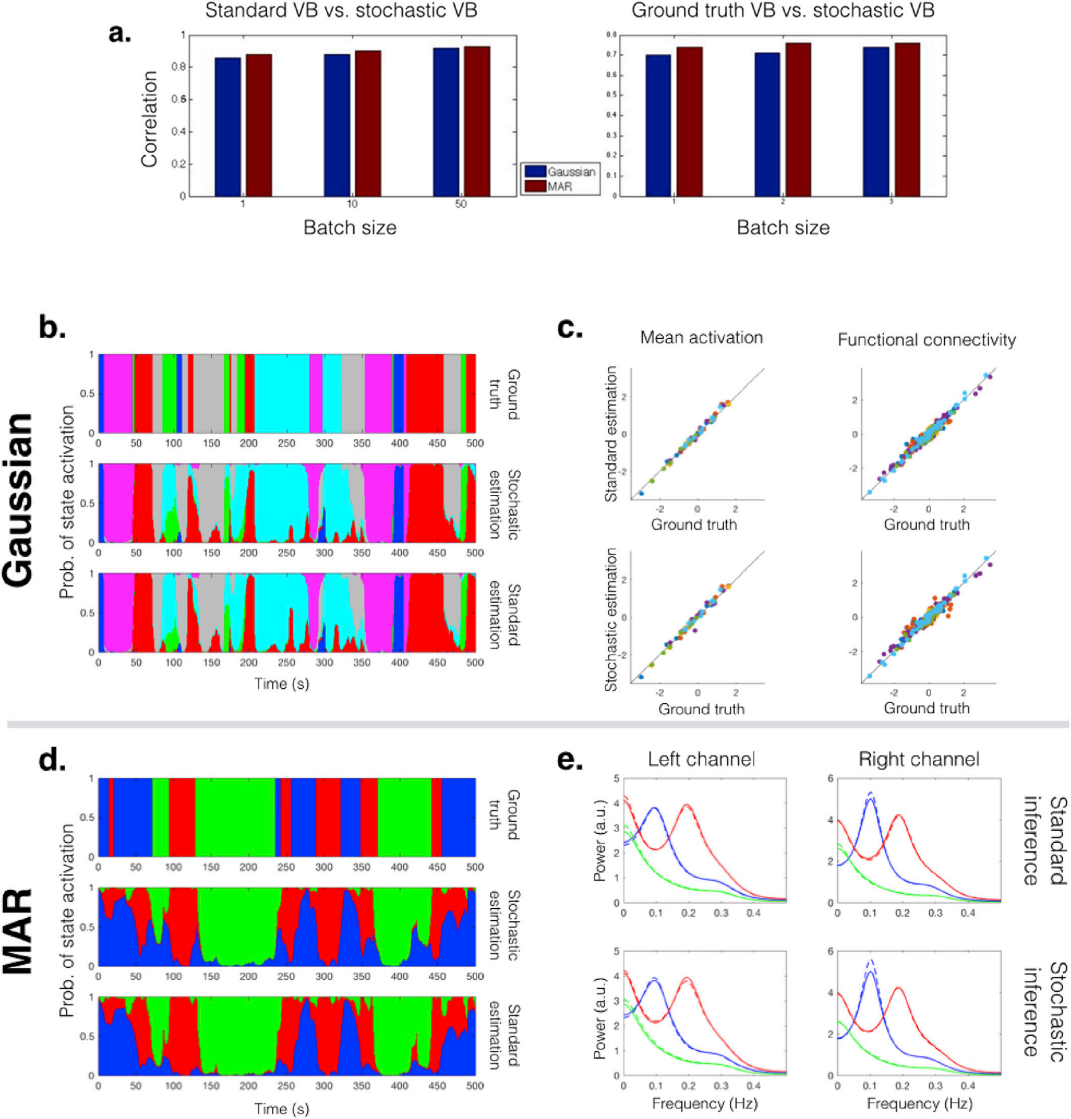
Results for simulated data, for the Gaussian and the MAR case. **(a)** Mean correlation between state time courses inferred using the non-stochastic approach and the stochastic approach (left), and between state time courses inferred using the stochastic approach and the ground truth (right), for the Gaussian observation model with two different perturbation sizes and the MAR observation model, for different batch sizes. **(b)** Ground truth and inferred state time courses for one subject, using the Gaussian observation model with high intersubject variability. **(c)** Mean activation (mean of the Gaussian distribution) and functional connectivity (off-diagonal elements of the covariance matrix) of the ground truth model against the standard and stochastic inference estimations; each colour represents a different state, and each dot represents a region if showing the mean or a pair of regions if showing functional connectivity. **(d)** Ground truth and inferred state time courses for one subject, using the MAR observation model. **(e)** Power estimation for each channel (left and right) and each state (blue, red and green) for the ground truth model (continuous lines) and the estimated models (discontinuous lines; top panels correspond to standard inference and bottom panels correspond to stochastic inference).

The aim is to use the simulated data to verify that the stochastic algorithm's performance is consistent with the standard HMM inference, by comparing model inference using the non-stochastic and stochastic algorithms. We ran (standard) non-stochastic inference and stochastic inference for 100 different random state time courses. Because the ordering of the states in the output is random, we matched the states of the estimation and the ground truth model based on the correlations between the state time courses. Once the states are matched, we use the average correlation between matched states as a similarity measure to compare the estimations to the ground truth, and to compare the standard inference to the stochastic inference. Fig. 2**a** shows, for three different stochastic batch sizes, the mean correlation between the stochastic and non-stochastic algorithms (left) as well as the mean correlation between the stochastic estimations and the ground truth (right). A full representation of the 100 runs is presented in the form of histograms in Fig. SI-1. In general, even in the limit of a batch size of one subject, we find the stochastic algorithm still infers state time courses that are well correlated with the non-stochastic algorithm. As expected, however, the similarity between the two algorithms is higher for larger batch sizes (in the limit of a batch size equal to the number of subjects, the two algorithms are exactly equivalent).

Representative examples of the inferred state time courses for a single subject are shown in Fig. 2**b** and **d** for the Gaussian and the MAR cases respectively (these examples' accuracies correspond to the median of the 100 runs). The ground truth average activity (mean of the state's Gaussian distribution) and functional connectivity (off-diagonal elements of the state covariance matrices) for the Gaussian case is plot against the standard and stochastic estimations (with batch size equal to 50) in Fig. 2**c**, where each colour represents a different state, and each dot represents a region (if showing the mean) or a pair of regions (if showing functional connectivity). The state spectra information for the ground truth (solid lines) versus the standard inference and stochastic inference estimations (discontinuous lines) is illustrated in Fig. 2**e** for the two channels. These results together indicate that the inference is consistent between the standard and the stochastic algorithms for a variety of configuration parameters, with both algorithms able to reasonably recover the ground truth.

### 3.2 HCP resting fMRI data

We used stochastic inference on 820 resting-state fMRI subjects from the HCP to obtain 12 states of quasi-stationary brain connectivity. We ran the algorithm 5 times, with an average running time of 221min (minimum and maximum were 208min and 225min) using a standard workstation (endowed with four Intel Xeon CPU E5-2643 0 3.30 GHz processors). We used *M* = 30. Some of the results that follow are from a selected run of the stochastic algorithm. However, the different runs were relatively similar (see below). With these results, our goal is to illustrate (i) the type of information the HMM can provide about brain dynamics, and (ii) the ability of the stochastic inference to produce useful and non-trivial results. Note that it is not possible to make a comparison of the standard inference and the stochastic inference in this case because the standard inference is computationally implausible given the size of the data set.

**Fig. 3:**
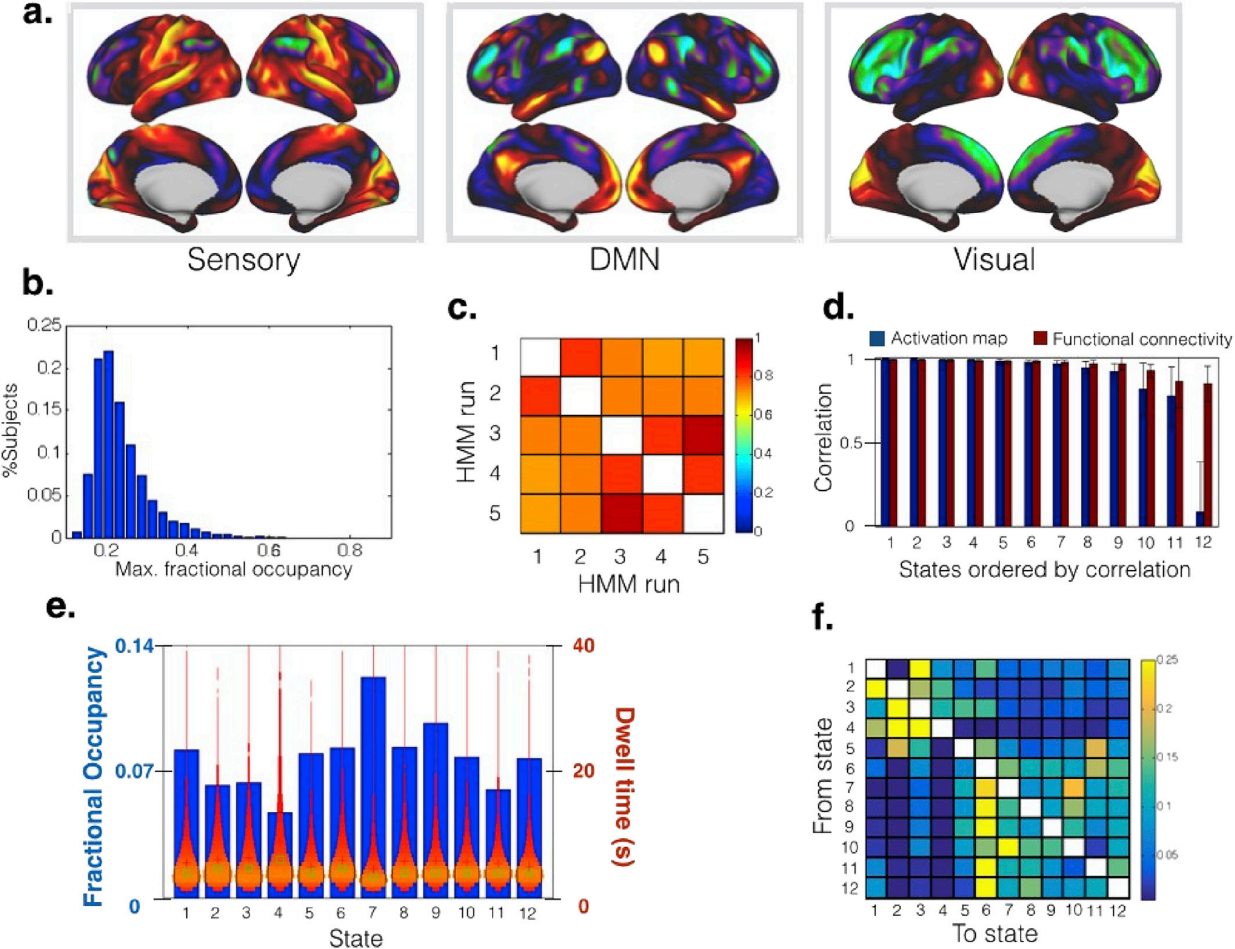
Results of the stochastic HMM inference on resting-state fMRI data from 820 HCP subjects, using a Gaussian distribution to describe each state. (**a**) Mean activation for three example states. (**b**) Histogram of the maximum fractional occupancy, a measure aiming to check whether the HMM is able to characterise the dynamics of the data (see Results). (**c**) Correlation of the state time courses across different runs of the algorithm, showing that the results are robust and consistent across runs. (**d**) Correlation of the activation maps and functional connectivity between estimations obtained from separate half-splits of the data set, averaged across 5 random splits, and with the states ordered from less to more correlated. (**e**) Fractional occupancy (defined as the total time spent by the subjects in each state) and distribution of dwell time (i.e. the time spent in each state visit) per state, reflecting basic aspects of the temporal dynamics of the data. (**f**) Transition probability matrix, reflecting the probability to transition between every pair of states.

Fig. 3**a** shows the mean activation maps for three of the twelve networks (states), representing a sensory state, a default mode network (DMN) state, and a visual state (the rest are presented in Fig SI-2). Regarding the temporal information, the most fundamental output is the state fractional occupancy, defined as the proportion of time that each subject spends in each brain state. A model that captures the within-subject temporal dynamics effectively (as opposed to a model that only finds between-subject differences) would be expected to have subject-specific fractional occupancies such that single states do not “dominate” entire subjects. In other words, for an HMM to be useful in describing brain dynamics, we expect each subject's time to be shared by various states. A statistic reflecting the satisfaction of this minimum requirement is the maximum fractional occupancy; that is, for each subject or scanning session, how much time of the time series is taken by the state that takes the longest. Fig. 3**b** shows a histogram with the maximum fractional occupancy. Most subjects have maximum fractional occupancy below 0.4, demonstrating that most subjects need several states to be optimally described.

Another important sanity check is how consistent are separate runs of the stochastic inference. This is important, because the standard HMM estimation is, as mentioned earlier, known to be to some extent dependent on the initialisation, and our algorithm is introducing an additional stochastic factor. To investigate this, we match the states across different HMM stochastic estimations (using the Hungarian algorithm from Munkres (1957), applied to the correlation of the state time courses), and collect the correlation of the state time courses between the re-ordered estimations (i.e. for each pair of matched states from two different runs, we get the correlation between these states' time courses). Fig. 3**c** shows that these correlations are high (most typically over 0.75), reflecting the robustness of the proposed stochastic inference approach.

A further robustness test, which would also speak to the reliability of the potential scientific conclusions, is to split the data set into two halves and run the HMM separately. Here, we have used 5 different half-splits, computing the correlation between the activation maps and functional connectivity (off-diagonal elements of the covariance matrix) between the two 410-subject HMM estimations for each of the splits. Fig. 3**d** shows these correlations (averaged across the 5 splits) for each state, with the states ordered from more to less correlated. Most states show a high correlation between the two half-split estimations for both mean activation and functional connectivity, with the exception of one state, which has a relatively low correlation for the activation maps. This state, however, has a mean activation very close to zero in both estimations, with the covariance matrix instead capturing most of the distinct state-specific characteristics of the data when the state is active. Furthermore, Fig SI-4a examines the stability of the transition probabilities between half-splits, which are also quite robust.

Having assessed the basic validity of the stochastic inference, we analysed in more detail the temporal characteristics of one of the runs. Fig. 3**e** shows the fractional occupancy for every state along with the distribution of the states' dwell times (which refers to the temporal duration of the state visits). From this figure, we can see certain differences in the sense that some states are visited more than others. However, the differences in terms of dwell times are small. Finally, Fig. 3**f** shows the transition probability matrix, showing that some (state-pair) transitions are more probable than others (Allen et al., 2014). Altogether, these results demonstrate that the HMM, when combined with the stochastic inference algorithm, can reproducibly model brain dynamics in large fMRI data sets.

### 3.3 Biobank resting fMRI data

**Fig. 4:**
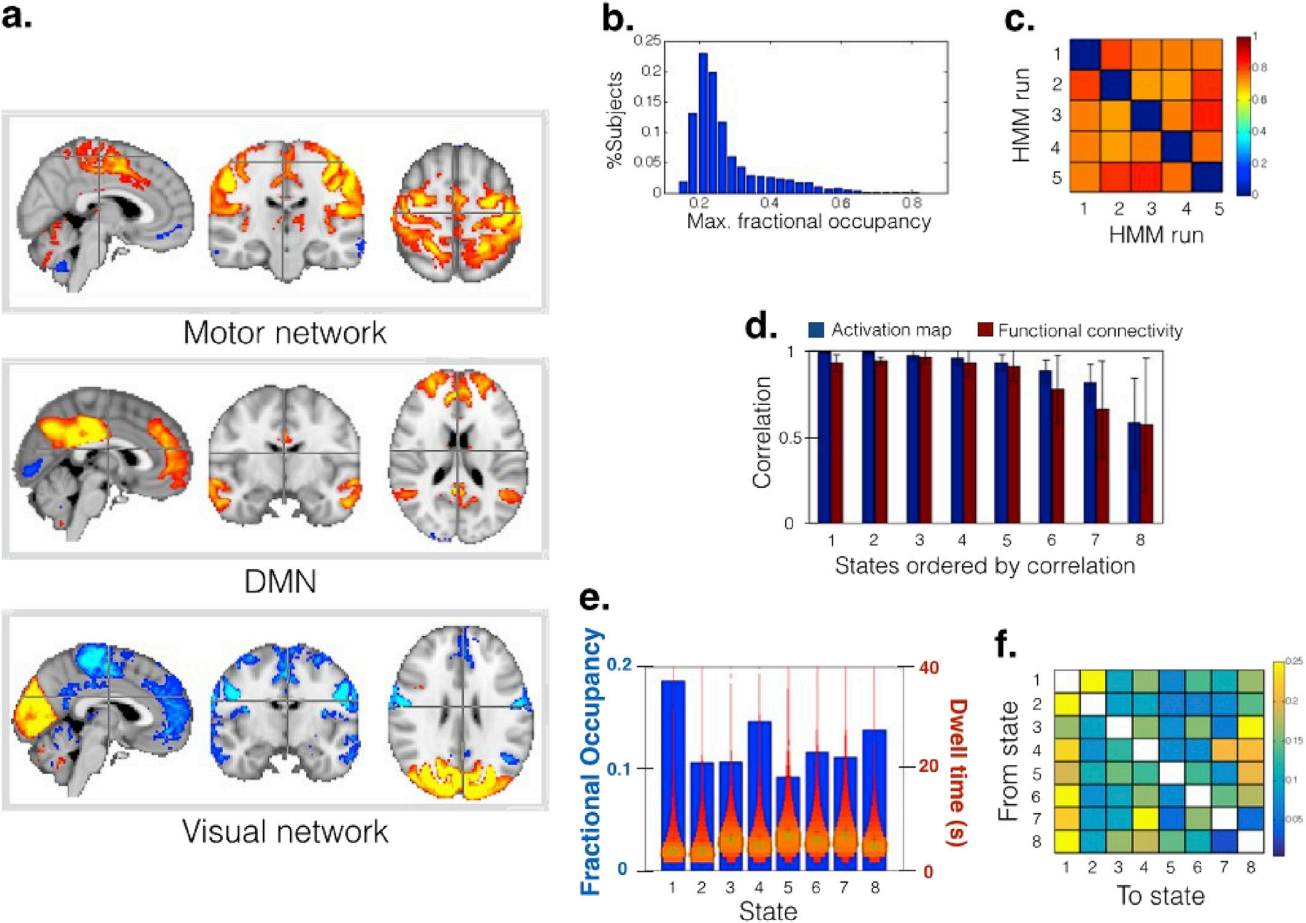
Results of the stochastic HMM inference on resting-state fMRI data from 5248 UK Biobank subjects, using a Gaussian distribution to describe each state. The description of each panel is analogous to that of Fig. 2, except for the activation maps being volumetric instead of showing the cortical surface.

Next, we used the stochastic algorithm on 5248 fMRI subjects from the resting-state data set of Biobank, again performing 5 runs of the algorithm with *M* = 250. The average running time was 144min (minimum and maximum were 141min and 148min). In this case, tests based on half splits and 12 states (see *HCP Resting fMRI Data* section) revealed that 8 out of 12 states were robust across halves (see Fig SI-5). The HCP data set allowed 12 reliable states possibly because of the higher data quality and more scanning time per subject. Basing then on the reliability of the results, we present here the model with 8 states. Three of them, representing sensory-motor, DMN and visual networks, are displayed in Fig. 4**a** (the rest are shown in Fig SI-3). The rest of the figures (Fig. 4**b-f**) are analogous to the HCP results, and the conclusions are similar. The HMM can capture the dynamics of brain activity (Fig. 4**b**), and the stochastic inference was robust and consistent across different runs of the algorithm (Fig. 4**c**) and between random half-splits of the data set (Figs. 4**d** and SI-4b); this consistency was however less pronounced than in the HCP data set, probably due to the very high quality of the HCP data. As with the HCP, there were some differences in fractional occupancies across states. In this case, however, the differences in dwell time were larger than for the HCP, but still not huge.

It is worth noting that, although some similarities exist, there are certain differences between the HCP and the Biobank states. This is possibly due not only to differences in the pipeline and the characteristics of the data, but also to fact that the data is projected into different spaces: whereas the state maps for Biobank are volumetric, the HCP maps refer to the cortical surfaces. Indeed, the ICA decompositions emphasise different areas in the two data sets. To illustrate this discrepancy, Fig. SI-6 shows “representability” maps, where, for each voxel (or grayordinate), we compute the sum of the absolute values of all ICA components. This indicates how much is each region represented by each ICA decomposition. These apparent differences in the independent components on which the HMM was run are likely to explain much of the differences in the HMM results.

### 3.4 Task MEG data

Finally, we test the performance of the stochastic inference algorithm in task using 52 MEG subjects from the HCP. In this case, we used a MAR observation model of order 5 to describe the states, such that, as discussed above, the segmentation will be based on the spectral information of the data. Given the relative simplicity of the task and because we have only two data channels (brain regions), we limit our HMM to infer 4 states only. Again, we run the algorithm 5 times with *M* = 8, which took 166min on average (with minimum and maximum of 90min and 229min). Note that the size of data set and the number of model parameters just about permits the use of the standard inference approach, albeit at great computational cost. The stochastic algorithm can relatively easily handle this data and model, and, although we here focus on only two regions to allow for straightforward comparisons with previous work (Vidaurre et al., 2016), it has the potential to be extended, for example, to work over more brain regions (e.g. spanning the whole brain).

**Fig. 5:**
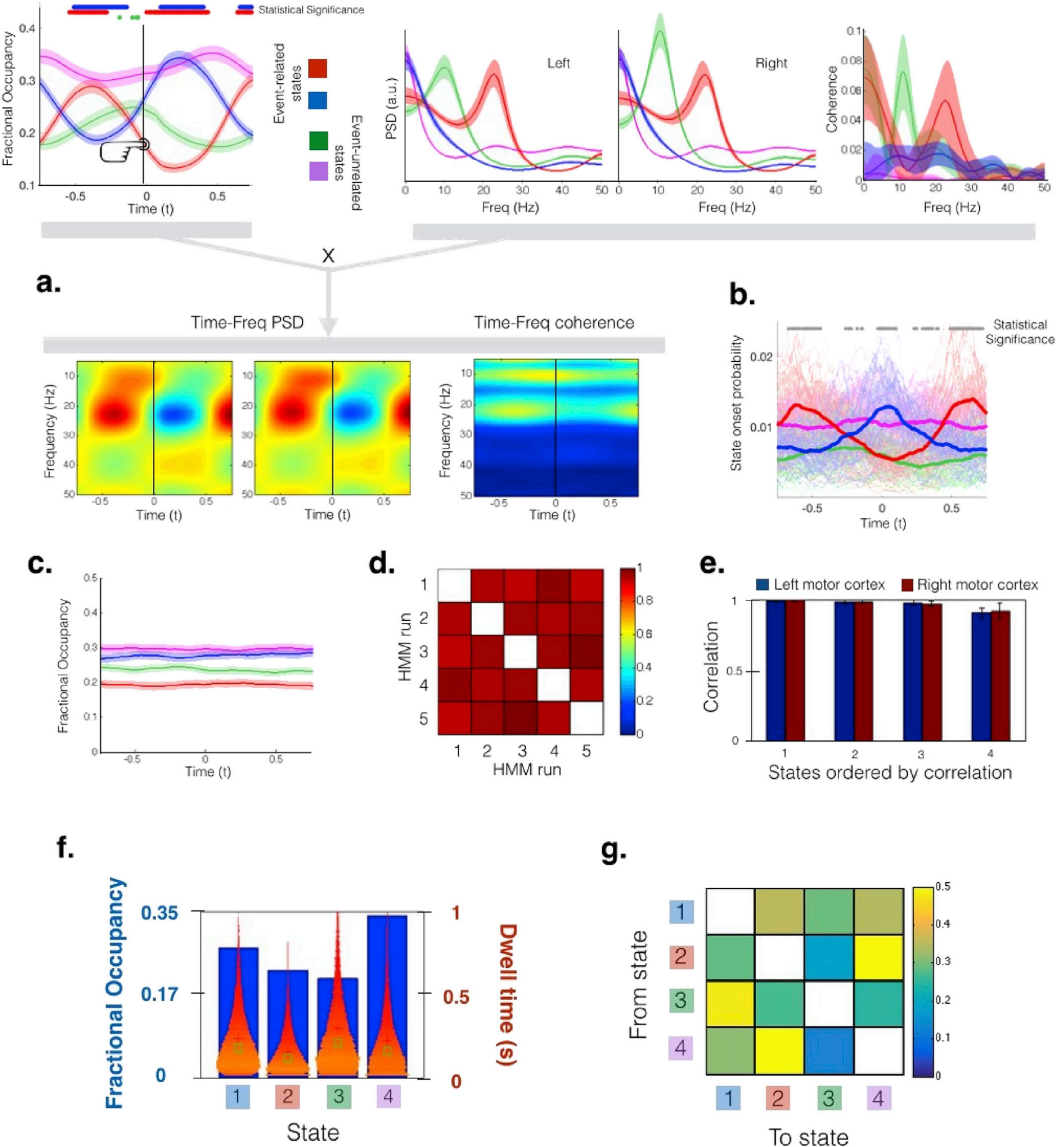
Results of the stochastic HMM inference on task MEG data from 52 MEG subjects, modelling the activity of the two motor cortices during a button-press task (1.2s inter-trial interval). To describe each state, we used a multivariate autoregressive model (MAR) distribution that can capture the spectral properties of the data. (**a**) The HMM, blind to the task information during the inference of the model, can discover states that reflect the underlying task dynamics; correspondingly, the state evoked response (top left panel) differentiates between event-related components that increase in probability (e.g. state blue, representing the evoked response) and others that show a decrease (e.g. state red, representing the event-related desynchronisation). When combining each state's temporal (top left panel) and spectral information (top right panel), the approach provides a time-frequency description of the data (bottom panel). Notably, the HMM captures the rhythmicity of the task (i.e. hand movements every 1.2s). **(b)** State onset probability, indicating, for each state and time point, the proportion of trials for which the state *becomes* active at this time point; each thin line represents a subject and the thick line is the group average; statistical significance of whether the dominant state has significantly larger onset probability than any of the other states is shown on top. (**c**) State evoked response for data where no task is performed. (**d**) Correlation of the state time courses across different runs of the algorithm, proving that the runs are extremely consistent. (**e**) Correlation of the power spectral densities between estimations obtained from separate half-splits of the data set, averaged across 5 random splits, and with the states ordered from less to more correlated. (**f**) Fractional occupancy and dwell times of the four states. (**g**) Transition probability matrix.

We next show that, similar to what was previously shown using standard inference (Vidaurre et al., 2016), the HMM can capture differences in activity modulated by the task. Fig. 5**a** (top left) shows the average state time courses (with standard errors) around the button press event, which can be interpreted as a “state evoked response”, i.e. what is the average probability (across trials) of each state being active at each time point. Two states, blue and red, capture most of the task-relevant dynamics. This is confirmed quantitatively by applying statistical testing, where we tested for each state and time point whether the fractional occupancy of the state is significantly different (higher or lower) at this time point than in the rest of the trial (permutation testing, significance level of 0.01). The results of these tests are depicted on top of Fig. 5**a**. It can be observed that it is mostly the red and the blue states that exhibit differences across time, suggesting that they are modulated by the task. Note that, unlike the study in Vidaurre et al. (2016), the button presses are here rhythmic and more frequent, possibly producing an entrainment of cortical activity to the movement. This effect, due to the neural activity of the previous button press “leaking” into the next trial, is expressed by the HMM as an oscillatory behaviour of the state time courses.

Fig. 5**a** also shows the spectral properties of the states, in terms of power (top centre) and coherence (top right). As expected, the estimation of coherence is noisier than that of power. Interestingly, the red state, which represents a strong activation in the beta band in both hemispheres, does not get depressed at the same time of the button press, as might be expected, but a bit later. Again, this is probably due to the resonating activity from the last button press, which was bringing a post-movement increment in beta activity when the new movement was starting. Temporal and spectral information can be combined to produce HMM-regularised time-frequency representations of the activity. This is shown in Fig. 5**a** (bottom).

As an alternative view to the fractional occupancy, we also looked in Fig. 5**b** at how the onset of the states (i.e. the instants when a state becomes active) relates to the task. The onset of the states can be interpreted as a point process, with one event per state occurrence indicating the times when this state becomes active. Hence, the *Onset Probability* measure reflects, for each time point and state, the percentage of trials for which this state becomes active precisely at this time point. Each light line corresponds to one subject, and the thick lines represent the mean across subjects. For visualisation purposes, the curves were smoothed. At the top of Fig. 5**b**, each grey dot indicates that the dominant state at this time point has significantly larger onset probability than any of the other states (permutation testing, significance level of 0.01), suggesting that it is the red and blue states that primarily attain significance. This figure gives a complementary perspective of the state dynamics. For example, it reveals that the blue state becomes active, on average, at exactly the time of the button press, reaching a peak of fractional occupancy at ∼+0.25s (Fig. 5**a**), and yielding to the red state slightly later than +0.5s.

Fig. 5**c** shows the average state fractional occupancy (with standard errors) for an HMM estimation obtained from the MEG resting-state data, where no movement is performed, and trials were artificially assigned to the resting-state session. Here, the states do not show any oscillatory pattern or any other consistent temporal variation (i.e. the average state time courses are flat across the entire trial, and the only difference between states in terms of the global temporal information is the total average fractional occupancy across all time points). Finally, Fig. 5d and **e** shows that the estimations are very consistent across different runs of the algorithm and between random half-splits of the data set (for the half-splits, the correlations are computed on the power spectral density obtained from the MAR coefficients). Additional temporal information for one of the runs is presented next, showing fractional occupancy and dwell time distribution (Fig. 5**f**), and the transition probability matrix (Fig. 5**g**).

## 4 Discussion

Current efforts are being undertaken to publicly release large data sets with hundreds or even thousands of subjects. Also, improvements and standardisation of protocols, together with the increase of widespread data sharing, will likely make it possible to combine and fuse neuroimaging data from different recording modalities. The availability of more data allows for more sophisticated analyses that will allow us to ask particular questions for the first time. For these reasons, it is crucial to have methods that can be scaled to large data sets. In this paper, we have proposed a method that can leverage large data sets' availability to address a difficult question: the characterisation of dynamic connectivity in both task and rest.

The HMM methodology has been proven useful in the past (Baker et al., 2014, Vidaurre et al., 2016, Vidaurre et al., Woolrich). In resting, the dynamics of whole-brain resting state networks have been characterised in MEG (Baker et al., 2014) and fMRI (Vidaurre et al., Woolrich), unveiling, for example, that the transitions between states or networks are far from random. In task, the HMM was able to find transient, short-lived states with specific spectral signatures that meaningfully characterised the primary motor cortex dynamics during a simple motor task. Here we have substantially enhanced the method to allow for the characterisation of dynamic brain networks in both task and rest from very large to very large datasets.

The HMM provides a rich description of brain network dynamics, without the limitations of sliding-window approaches. More specifically, as the number of inferred HMM states grows we are potentially able to capture most of the information contained in the data without being bound to a predefined time-scale (the width of the window) and without any of the estimation problems of the sliding window approach (i.e. the uncertainty of an estimation based on just a few data points). For example, as the Biobank data set grows towards the goal of 100,000 subjects, with the proposed stochastic inference approach, we are confident to estimate HMMs with more states while still having more than enough data to estimate each state sufficiently well. Two interesting developments that would boost even further the potential of the data could be (i) the combination of models from different data sets using some principled quantitative approach, and (ii) the possibility of estimating a joint model using different data sets in a single inference process. This would require sufficiently uniform preprocessing pipelines and a mapping of the data to a common brain space (which is not currently the case for the HCP and UK Biobank).

While our method can be readily applied to both rest and task data, the study of functional dynamics in resting-state is however not free of controversy. For example, Laumann and colleagues (Laumann et al., 2016) reasonably argue that most of the observed changes in functional connectivity are due to factors other than true switches in cognitive content, namely head motion, sleep and sampling variability. Provided head motion is handled with care, and acknowledging the influence of sleep and drowsiness (undeniably a genuine neuronal and cognitive process that can be an issue in any analysis, whether on dynamic functional connectivity or otherwise), the main obstacle to identifying fast cognitive changes in resting-state data is the lack of statistical power of the sliding window approaches: because windows are relatively short, the observed changes are mostly driven by the high variance of the estimator (Hindriks et al., 2016). Here, we claim that studying dynamical functional connectivity in resting-state data is not necessarily an ill-posed problem; rather, the increasing availability of data makes possible to approach this problem convincingly, if we have to tools to tackle it. A strong argument supporting this claim is the demonstrated robustness of the method; in particular, if we split any of the three considered data sets into two halves and obtain a separate estimation for each half, the two estimations are highly consistent (see Results).

Note that the contribution of this paper is primarily targeting the computational issues of having a large amount of data in time, sessions and/or subjects. A related but different problem is the case when we have a large number of regions of interest. Unfortunately, the stochastic inference procedure would only address the computational aspects of it and not the overfitting issues. The stochastic scheme can be however useful when there is the possibility of collecting more data. Obviously, using more data will reduce overfitting and increase the computational cost, which can in turn be alleviated using the proposed stochastic inference scheme.

In summary, the approach presented here opens the door to new methodological research based on the HMM. For example, the combination of many different models to obtain an *ensemble* that perform much better than its parts has been a successful story in supervised learning (Hastie et al., 2003). Here, in an unsupervised learning scenario, we can leverage the computational efficiency of the algorithm to obtain a numerous set of HMMs that can somehow be combined to provide a richer description of the data. How these models can be combined, and what new aspects of data we could investigate by using such a coalescence of individual HMMs (potentially with different parametrisations), will be the subject of future investigations.

## Footnotes

1 https://github.com/OHBA-analysis/HMM-MAR.

2 https://www.humanconnectome.org/documentation/MEG/MEG1_Release_Reference_Manual.pdf.
