# Supplementary material for: Discovering dynamic brain networks from big data in rest and task
Source: Neuroimage. 2018 Oct 15;180(Pt B):646–56. doi: 10.1016/j.neuroimage.2017.06.077 (PMC6138951; doi:10.1016/j.neuroimage.2017.06.077)
Supplement: Supplementary file 1 [file mmc1.docx]

**Supplementary material**

**Initialisation of stochastic algorithm**

An important aspect HMM inference is the initialisation. The optimisation criterion might contain several minima, and that can potentially be aggravated when trained on large data sets. Although the noisy nature of the stochastic updates can help to circumvent shallow local minima, that still does not guarantee a good solution. For this reason, having an appropriate initialisation strategy is relevant to the performance of the method. Here, we run the standard variational inference approach on a subset of subjects at a time and compute the sufficient statistics per state, which, in the Gaussian case, reads

(6) *S_k_ = Σ_t_ Pr(s_t_=k) (Y_t_’ Y_t_)* and *m_k_ = Σ_t_ Pr(s_t_=k) Y_t_*

with *k* indexing the states. Along with the state sufficient statistics, we also accumulate the sufficient statistics for the state transitions and initial probabilities, which, respectively, correspond to:

(7) P_lk_ = *Σ_t_ Pr(s_t_=l) Pr(s_t+1_=k)* and *π_k_ = Pr(s_1_=k)*

Subset after subset, we keep aggregating the sufficient statistics into a set of group sufficient statistics. Considering that the ordering of the HMM states is arbitrary, every time a new subset of subjects is estimated, we need to reorder (before aggregating) its states so that they match as closely as possible the group states. We compute the best reordering using the Hungarian algorithm [25] and the (symmetrised) Kullback-Leibler divergence as a measure of distance between the subset and the group distributions. These distributions can be straightforwardly derived from the sufficient statistics in Equation (6). Once all individual subsets have been processed, the accumulated group sufficient statistics naturally yield the initial parameters for both the distribution of the states and the transition (and initial) probabilities, so that we can start the process of stochastic inference.


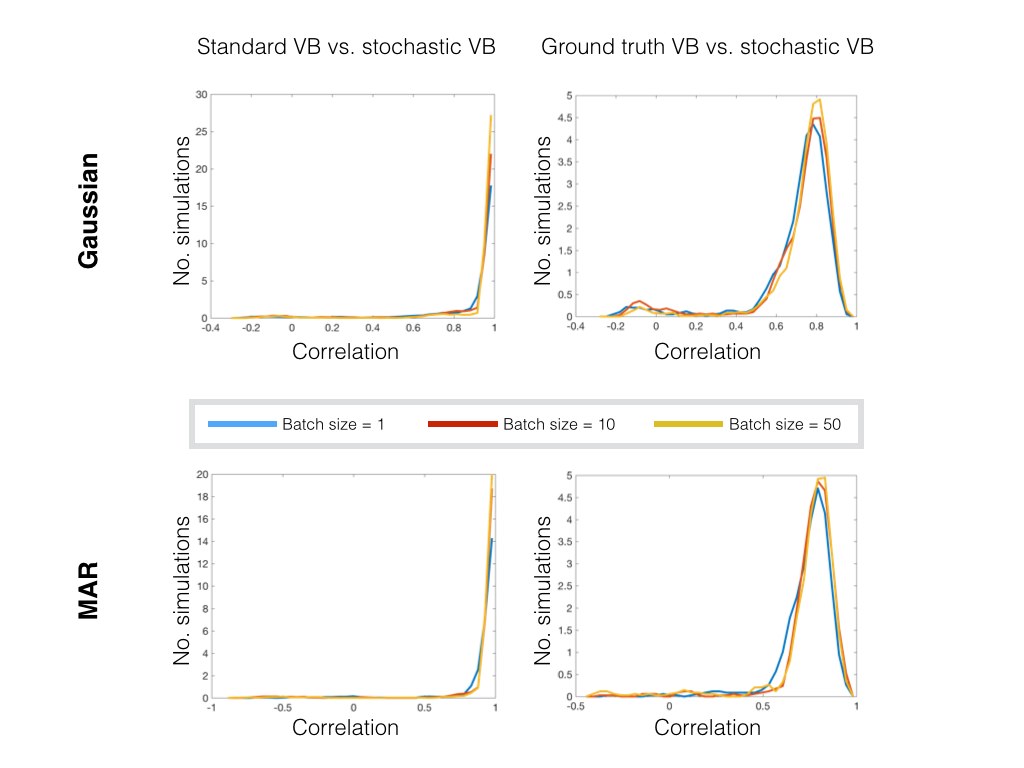
**Fig SI-1.** Detailed results for simulated data, for the Gaussian and the MAR case. Histograms reflect the correlations between the state time courses inferred using the non-stochastic approach and the stochastic approach (left), and between state time courses inferred using the stochastic approach and the ground truth (right), for the Gaussian observation model (top) with two different perturbation sizes and the MAR observation model (bottom). Occasionally, the HMM represents a single true state using two inferred states with ~50% probability each. In these cases, when evaluating the model, one of the inferred states is matched with the true state, and the other inferred state will be matched to a different state that the HMM did not successfully infer. This causes a small concentration of correlations in the histogram at slightly negative correlation values. This effect, however, does not occur for the MAR observation model.


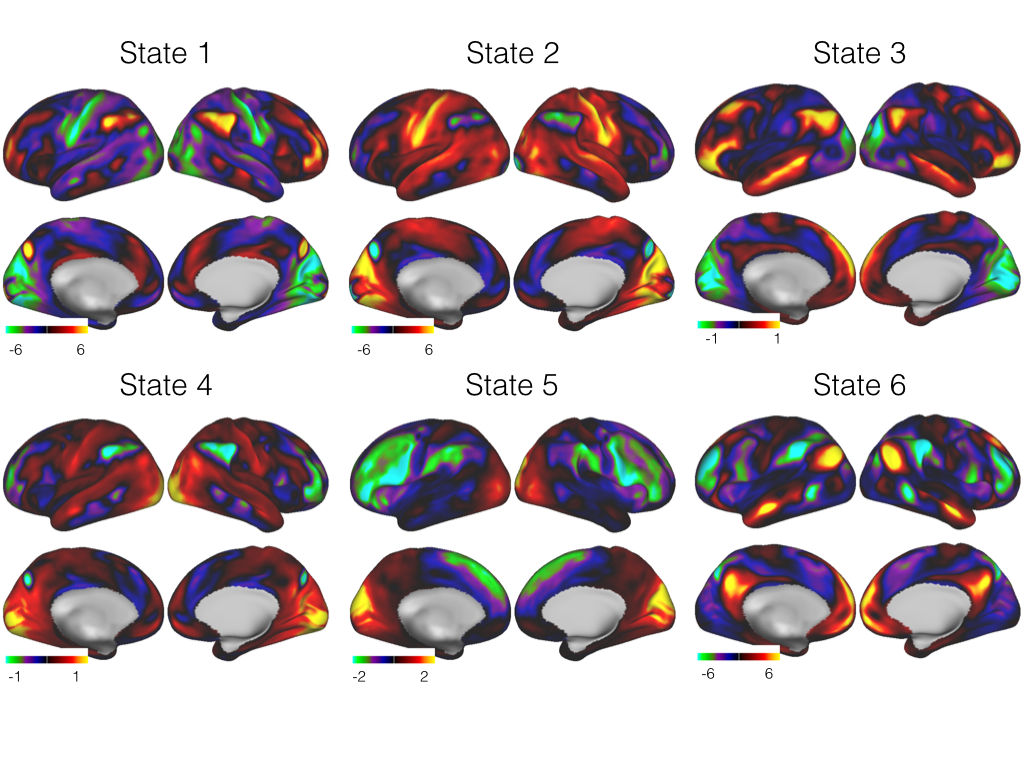


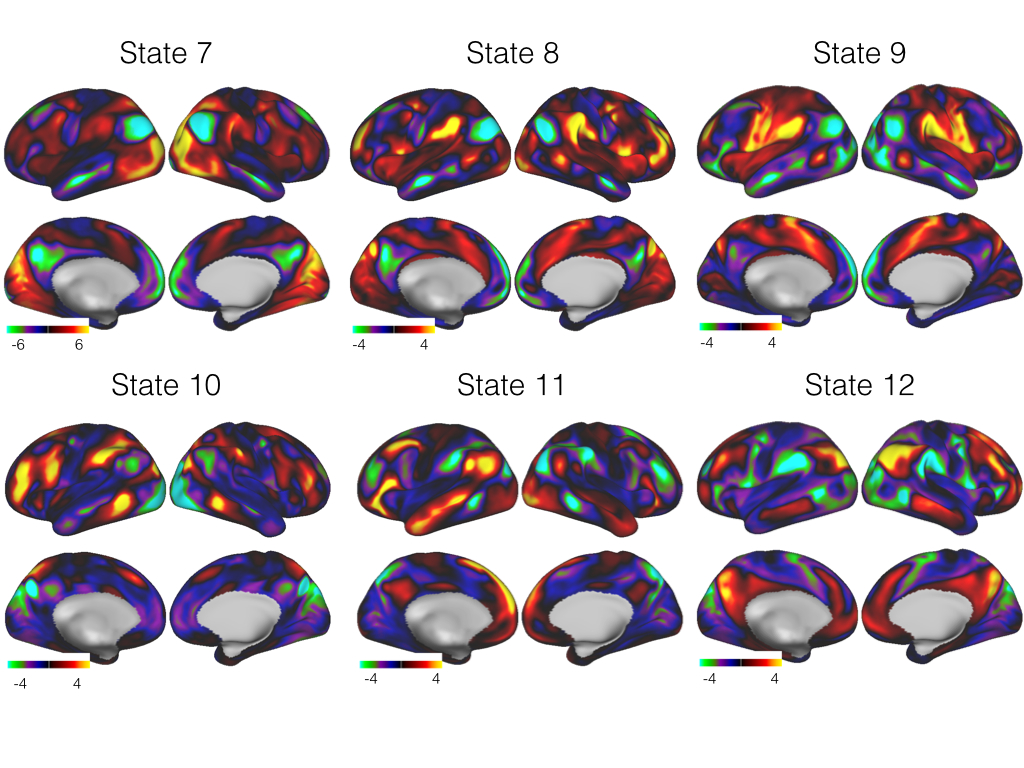


**Fig SI-2**. Mean activation for the twelve HMM states obtained from the fMRI resting-state HCP data set.


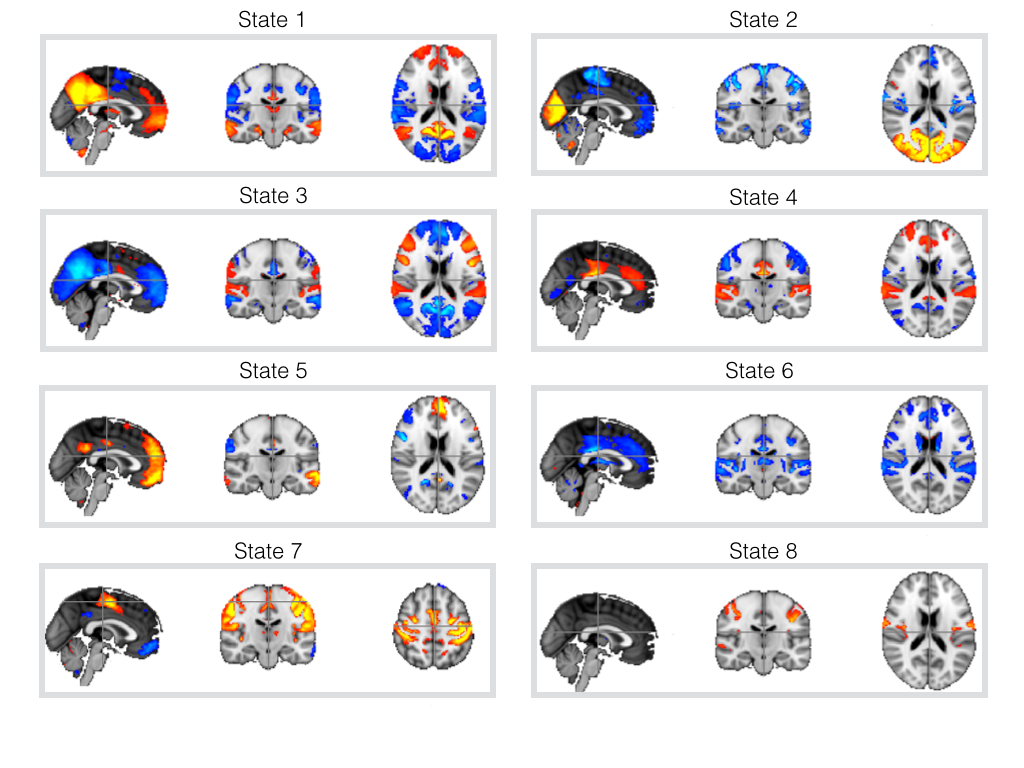


**Fig SI-3**. Mean activation for the twelve HMM states obtained from the fMRI resting-state Biobank data set.


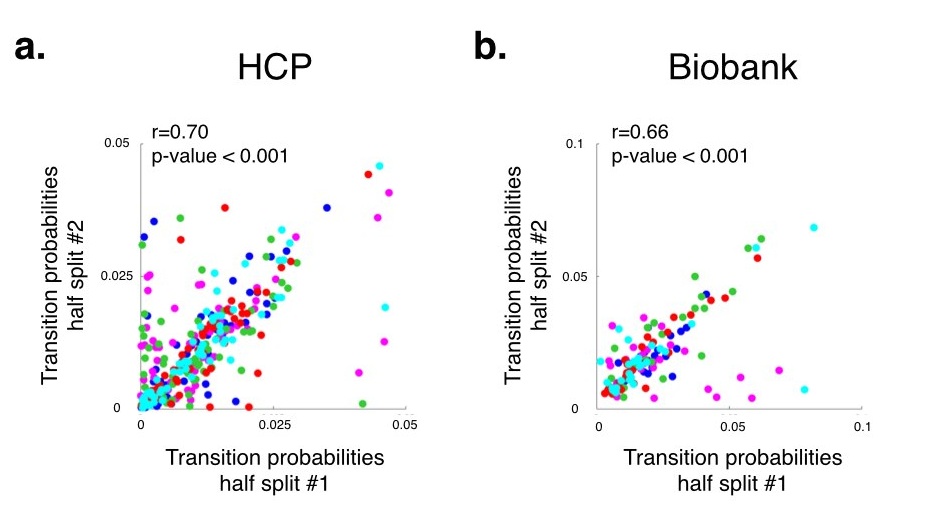


**Fig SI-4**. Correlation of the transition probability matrices obtained from five pairs of half-split runs for the HCP **(a)** and UK Biobank **(b)** data sets, where we randomly separated each data set into two halves and ran the HMM on each half separately. Each colour represents one of the five half-splits, and each dot corresponds to a transition probability between a pair of states. For each of the five half-splits, the states were matched between the two runs using a matching algorithm [25].


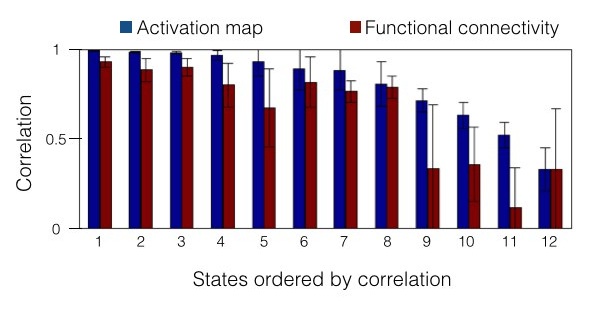


**Fig SI-5**. Correlation of the activation maps and functional connectivity between estimations obtained from separate half-splits of the Biobank data set using 12 instead of 8 states (see **Fig 4**). Results are obtained by averaging across 5 random splits, with the states ordered from less to more correlated. For each of the five half-splits, the states were matched between the two runs using a matching algorithm [25].


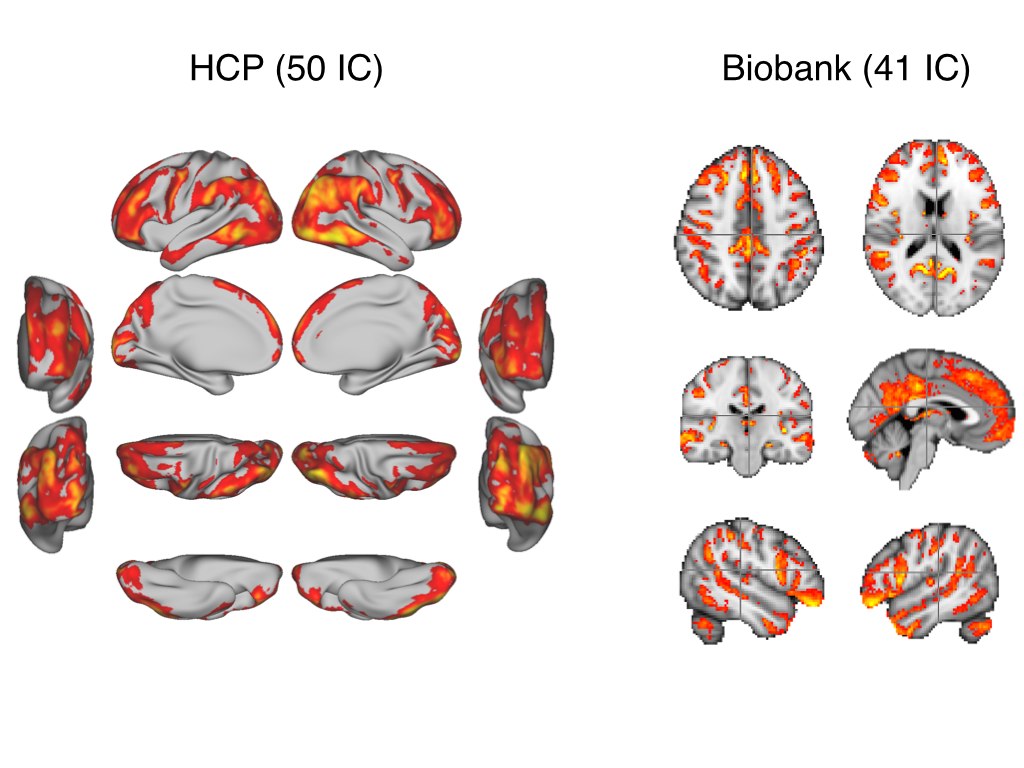


**Fig SI-6**. *Representability* maps reflecting the extent to which the ICA decompositions represent the different brain areas. For each grayordinate (left) or voxel (right), we computed the sum of the absolute values of all ICA components, such that a higher value (warmer colour) indicates that the area is better represented.
